# Pulsed electric field at resonance frequency combat *Klebsiella pneumonia* biofilms

**DOI:** 10.1007/s00253-024-13330-z

**Published:** 2024-11-05

**Authors:** Dorria H. Mohamed, Haitham Mohammed, Reem H. El-Gebaly, Mohamed Adam, Fadel M. Ali

**Affiliations:** 1 Biophysics Department, Faculty of Science, Cairo University, Giza, Egypt; 2 Agricultural Zoology and Nematology Department, Faculty of Agriculture, Cairo University, Cairo, Egypt

**Keywords:** Antimicrobial resistance, *K. pneumonia*, Extremely low-frequency, Pulsed electric field, Antibiotic susceptibility, Biofilm

## Abstract

Healtcare-associated infections have increased due to the development of antimicrobial resistance (AMR) of Gram-negative pathogens (GNPs) and the development of outbreacks over the past two decades. In this work, we investigated how exposure to positive electric pulses affects the growth characteristics of *Klebsiella pneumonia* (*K. pneumonia*), a common cause of pneumonia. We explored the impact of varying exposure frequencies (0.2–2 Hz) and time (15–90 min, at resonance frequency) on bioelectric signals produced during cell division, biofilm formation, and bacterial antibiotic susceptibility. Our research found that an extremely low-frequency pulsed electric field (ELF-PEF) significantly inhibited *K. pneumonia* growth. Specifically, exposure to 0.8 Hz for one hour increased the antibiotic susceptibility of *K. pneumonia* to inhibitors of cell wall formation, proteins, β-lactamase, DNA, and other substances. We also noticed a notable decrease in *K. pneumonia* biofilm development exposed to ELF-PEF. Our results suggest that the interaction of *K. pneumonia* cells with ELF-PEF at the specified frequency and time alters cellular activity and bacterial structure. This technique may be used in the future to treat *K. pneumonia* infections both in vitro and *in vivo.*

**Supplementary Information:**

The online version contains supplementary material available at 10.1007/s00253-024-13330-z.

## Key points

• *Extremely low-frequency pulsed electric fields inhibit the growth of K. pneumonia.*

•* ELF-PEF increases antibiotic susceptibility and reduces biofilm formation in K. pneumonia.*

• *ELF-PEF showed potential for treating K. pneumonia infections.*

**Supplementary Information:**

The online version contains supplementary material available at 10.1007/s00253-024-13330-z.

## Introduction

The phenomenon of antibiotic resistance is a global problem, and the World Health Organization (WHO) has designated *Klebsiella pneumonia* as one of the high-priority species (Kwiatkowski et al. 2022). For over 20 years, Egypt has experienced a high incidence of antimicrobial resistance (AMR) in Gram-negative pathogens (GNPs), which has led to an increase in nosocomial infections and fatalities (El-Kholy et al. 2020). Many diseases, including bloodstream infections, urinary tract infections, pneumonia, and infections of the skin and soft tissues are caused by this bacteria. (David et al. 2019; Domenico et al. 2020).

One of the most common GNP bacteria in the world, *K. pneumonia* is the cause of outbreaks associated with opportunistic infections in patients with compromised immune systems, including diabetics, alcoholics, and hospitalized patients with indwelling devices (Khan et al. 2010; Berrazeg et al. 2013; Al-Agha et al. 2017; Bouhrour et al. 2024). The extensive use of antibiotics in recent years has led to clinically isolated *K. pneumonia* becoming increasingly resistant to treatment. Extended-spectrum beta-lactamases (ESBL)-generating *K. pneumonia* and carbapenem-resistant *K. pneumonia* are significant factors contributing to the problem of antibiotic resistance (Nirwati et al. 2019; Domenico et al. 2020; Karimi et al. 2021). The formation of biofilms is a critical component of K. *pneumonia’s* pathogenicity, as they can transfer mobile components that are resistant to antibiotics, offer physical protection to bacteria, and increase their susceptibility to antibiotics, persistence, and dissemination (Cubero et al. 2019; Joseph et al. 2024). Biofilms are composed of extracellular DNA, proteins, and polysaccharides enclosed in bacterial clumps that resemble extracellular networks and can form on tissues, organs, and medical devices. Biofilms play a significant role in various diseases, such as chronic respiratory infection, chronic lung disease, chronic obstructive pulmonary disease (COPD), and ventilator-associated pneumonia (Boisvert, A. et al., 2016; Panickar et al. 2024) Most organisms in a biofilm are protected by the EPS layers and are resistant to various antimicrobial drugs (Singh et al. 2017; Mirghani et al. 2022).

Given the excessive and inappropriate use of antibiotics worldwide, alternative therapies, such as extremely low-frequency electromagnetic fields (ELF-EMF) are being investigated (Askaripour and Żak 2024). Previous studies have shown that ELF-EMFs can significantly impact cellular functions (Garip et al., 2011; Oncul et al. 2016; Guzmán-Armenteros et al. 2024), impact the rate of bacterial growth and viability (Fadel et al. 2014), and modify antibiotic susceptibility in a frequency-dependent way (Segatore et al. 2012; Woroszyło et al. 2021).

The purpose of this work is to examine how ELF- ELF-pulsed electric Fields affect *K. pneumonia* and to determine the resonance frequency that can inhibit the bacteria’s ability to divide and block its activity. Additionally, we aim to investigate potential modifications to viability, antibiotic susceptibility, and biofilm formation. Our findings could provide valuable insights into the potential of ELF-PEF as a treatment for *K. pneumonia* infections and could pave the way for novel in vitro and in vivo applications.

## Materials and methods

### Bacterial strains and growth conditions

In the present study, two *K. pneumonia* strains from the Central Public Health Laboratories and the Animal Health Research Institute (AHRI) collection were investigated. The strains were reliably identified as *K. pneumonia* (98%) using the VITEK 2 Compact system. *K. pneumoniae ATCC 13,883* strain was used as a benchmark for pneumonia as a reference strain and other isolates from the patient, To prepare the strains for each stage of the experiment, MacConkey agar was used for growing them (bioMérieux, Warsaw, Poland) and incubated aerobically at 37 °C for 24 h. Subsequently, two individual colonies of each strain of *K. pneumoniae* were selected and placed in a test tube containing 5 ml of sterile Tryptic Soy Broth (TSB) at pH 7.1 (Biolife, Milan, Italy). The test tubes were then incubated for 18 h at 37 °C to establish a subculture. A final concentration of 10^8^ cfu ml^−1^ (colony-forming units) was attained by inoculating 500 ml screw-capped flasks with 150 ml of sterile TSB media using this subculture. The cultures were maintained at 37 °C, with hourly interruptions for sample collection for absorbance measurement at a wavelength of 600 nm using a spectrophotometer (2602 UV/Vis; Jenway, Stone, Staffordshire UK) and sterile broth medium as a reference. Concurrently, the bacterial cell concentration (cfu ml^−1^) was determined using the viable plate counting technique (Fig.S1) (Skinner et al. 1952; Monod 2012). Each experiment was repeated four times, and the averages were computed. The cell count (cfu ml^−1^) and the sample absorbance at 600 nm were plotted on a standard count-absorbance calibration curve.

To create a square pulsed current with a 50% duty cycle and a range of frequencies for cell exposure, the study used electronic equipment that provided a 9 V-DC power supply (Ali et al. 2016). After converting the square pulses into a DC/DC voltage converter, a voltage field with an intensity of (400 ± 25) V was created. The suspension of the sample was sandwiched between two copper plates measuring 35 by 20 cm^2^ and 20 cm apart. As seen in (Fig.S2), the apparatus was constructed on-site at the German University of Cairo, Egypt’s physics lab following procedures outlined in previous reports by Serag (2013) and Serag et al. (2014).

## Inhibition frequency determination

To initiate a broth subculture, two bacterial colonies from a Tryptic Soy agar plate were introduced into a test tube containing 7 ml of sterile Tryptic Soy agar broth at pH 7.1 (Biolife, Milan, Italy). Subsequently, the subculture was placed in an incubator for 24 h at 37 °C. Following this incubation period, 7 ml of Tryptic Soy broth was sterilized and distributed into 24 test tubes. While most of the tubes underwent pulsed electric field (PEF) treatment for 30 min at intervals of 0.2 Hz, one tube served as a control. Throughout the exposure process, all tubes were maintained at room temperature. At intervals of two hours, samples were collected for absorbance measurements at a wavelength of 600 nm using a spectrophotometer, with sterile broth media as the reference. Subsequently, all tubes, including both exposed and control samples, were placed in an incubator at 37 °C. In Eq. (1), the inhibition percentage difference (D%) at the 24-hour mark was calculated for each sample compared to the control, and these values were averaged. The average inhibition D% data were then plotted against frequencies ranging from 0.2 Hz to 2 Hz to illustrate the resonance curve for the study. The experiment was replicated under similar conditions but with varying durations in the range of 15–90 min (at 15-minute intervals) at a fixed frequency of 0.8 Hz. The inhibition D% between each sample and the control at the 24-hour mark of incubation was determined using growth curves.

$$\:\mathrm{I}\mathrm{n}\mathrm{h}\mathrm{i}\mathrm{b}\mathrm{i}\mathrm{t}\mathrm{i}\mathrm{o}\mathrm{n}\:\mathrm{P}\mathrm{e}\mathrm{r}\mathrm{c}\mathrm{e}\mathrm{n}\mathrm{t}\mathrm{a}\mathrm{g}\mathrm{e}\:\mathrm{d}\mathrm{i}\mathrm{f}\mathrm{f}\mathrm{e}\mathrm{r}\mathrm{e}\mathrm{n}\mathrm{c}\mathrm{e}\:\left(\mathrm{D}\mathrm{\%}\right)=\frac{\:\:\mathrm{O}\mathrm{D}600\:\left(\mathrm{e}\mathrm{x}\mathrm{p}\mathrm{o}\mathrm{s}\mathrm{e}\mathrm{d}\right)-\mathrm{O}\mathrm{D}600\:\left(\mathrm{c}\mathrm{o}\mathrm{n}\mathrm{t}\mathrm{r}\mathrm{o}\mathrm{l}\right)}{\:\mathrm{O}\mathrm{D}600\:\left(\mathrm{c}\mathrm{o}\mathrm{n}\mathrm{t}\mathrm{r}\mathrm{o}\mathrm{l}\right)}\:x\:100.\:\:\:\:\:\:\:\:\:\:\:\left(1\right)$$

## Antibiotic susceptibility test

The susceptibility of the K. *pneumoniae* bacterial isolate to 13 different antimicrobial agents, namely Imipenem (IPM, 10), Tetracycline (TE, 30), Ciprofloxacin (CIP, 5), Azithromycin (AZM, 15), Ampicillin (AMP, 10), Aztreonam (AT, 30), Levofloxacin (LEV, 5), Vancomycin (VA, 30), Amoxicillin (AMX, 10), Gentamicin (CN, 10), Cefepime (FEP, 30), Streptomycin (S, 10), and Cefoxitin (CXN, 30) was determined using the disc diffusion test (Chang et al. 2015). The zone reading chart, discs, and susceptibility breakpoints for each antimicrobial agent used in the test were provided by CLSI, M100-S17 guidelines (Humphries et al. 2021). The antimicrobial susceptibility test was carried out following the National Committee for Clinical Laboratory Standards (CLSI) protocol (Kurokawa et al. 2022). Seven groups, each comprising three samples, were prepared for each strain of K. pneumoniae suspension. One group functioned as a control, while the remaining six groups were subjected to pulsed electric field (PEF) at various frequencies and durations. Subsequent to the exposure period, Mueller Hinton Agar (MHA) plates were inoculated with samples from both the exposed and control groups. After the inoculum had dried (approximately three to five minutes), the appropriate antibiotic discs were aseptically placed on the agar surface using sterile forceps. Each disc was gently pressed down to ensure contact. The plates were then incubated for 24 h at 37 °C to determine the diameter of each inhibitory zone, which was subsequently compared with the zone reading chart for interpretation.

## Biofilm formation assays

Following overnight cultures of the tested organisms, a loopful was transferred to 5 mL of Tryptic Soy Broth (TSB) with 1% glucose (Lab M Ltd, UK) and incubated at 37 °C for 48 h. Utilizing sterile 96-well flat-bottom polystyrene tissue culture plates with adaptations from prior studies (Wang et al. 2021; Kwiatkowski, 2022), each well was loaded with 200 µL of the bacterial solution (Sigma-Aldrich Co. LLC, USA). A negative control broth was included to assess sterility. The plates underwent a 48-hour incubation period at 37 °C. After the incubation period, 180 µL of 95% ethanol was introduced to fix the bacteria for 30 min, followed by gentle washing of any remaining planktonic cells three times with 200 µL of sterile saline (Stepanovic et al. 2000). Subsequently, the solution was subjected to 20 min of darkness after the addition of 180 µL of a 0.1% (w/v) crystal violet solution from Merck Life Science in Poznan, Poland. The wells were then rinsed three times with 200 µL of sterile saline. Absorbance values at 630 nm for each well were determined using a SynergyTM LX Multi-Mode microplate reader (BioTek, Winooski, VT, USA). To calculate the inhibition percentage between each sample and the control at the 48th hour of incubation, Eq. (2) was applied, and the mean was computed. The curve depicting the average inhibition percentage at a consistent effective frequency over various time points was plotted for analysis. The percentage of K. *pneumoniae* biofilm inhibition for different exposure periods was determined using the formula developed by da Silva et al. (2019) and Elsaid et al. (2023).

2 $$\mathrm{age}\;\mathrm{of}\;\mathrm{biofilm}\;\mathrm{inhibition}\;\%=1-\frac{\mathrm{OD}630\;\mathrm{of}\;(\mathrm{cells}\;\mathrm{exposed}\;\mathrm{to}\;0.8\;\mathrm{Hz}\;\mathrm{pulses})}{\mathrm{OD}630\;\mathrm{of}\;(\mathrm{unexposed}\;\mathrm{cells})}\times100$$

### Statistical analysis

SPSS Statistics (V. 28) was employed for the statistical analysis. ANOVA, or one-way analysis of variance, was used. Every pair-wise mean difference has been evaluated using post hoc Tukey’s technique. The mean ± standard deviation was used to express the results. A statistically significant difference was defined as a p-value of less than 0.05.

## Results

### In vitro growth characterization of k. pneumoniae

(Fig. S3) illustrates the growth curve characteristics of two strains of K. *pneumoniae*: ATCC13883-reference strain and the patient’s strain. The growth curve displays three distinct phases: the lag phase, the exponential (log) phase, and the stationary phase. The lag phase depicted in the figure represents a period during which bacterial cells acclimate to their new environment. In this phase, there is a limited increase in bacterial cell numbers, but the metabolic activity within the cells intensifies, leading to the rapid synthesis of cellular macromolecules, especially enzymes, in anticipation of the subsequent growth phase. The lag phase, as indicated in the figure, concluded after 8 h, transitioning into the exponential (log) growth phase where cell numbers increase steadily until the 13th hour. Following this, the stationary phase ensues, characterized by a balance between cell division and cell death, leading to a plateau in cell count.

### Growth characteristics and resonance frequency

**Fig. 1:**
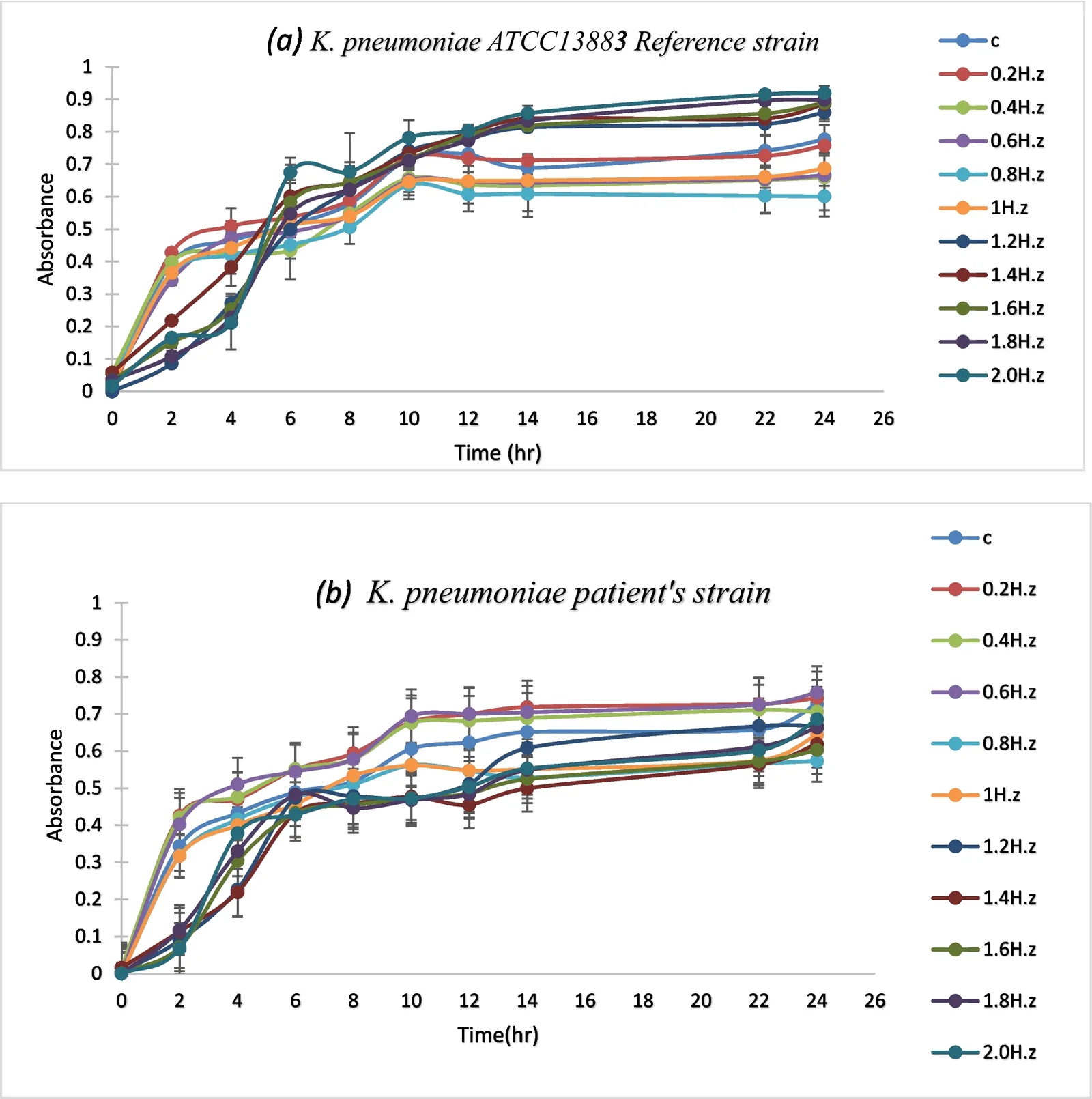
The growth curve of *K. pneumoniae* subjected to various frequencies PEF for a set duration of 30 min. (**a**) *K. pneumoniae ATCC13883-reference* strain (**b**) *K. pneumoniae* patient’s strain

**Fig. 2:**
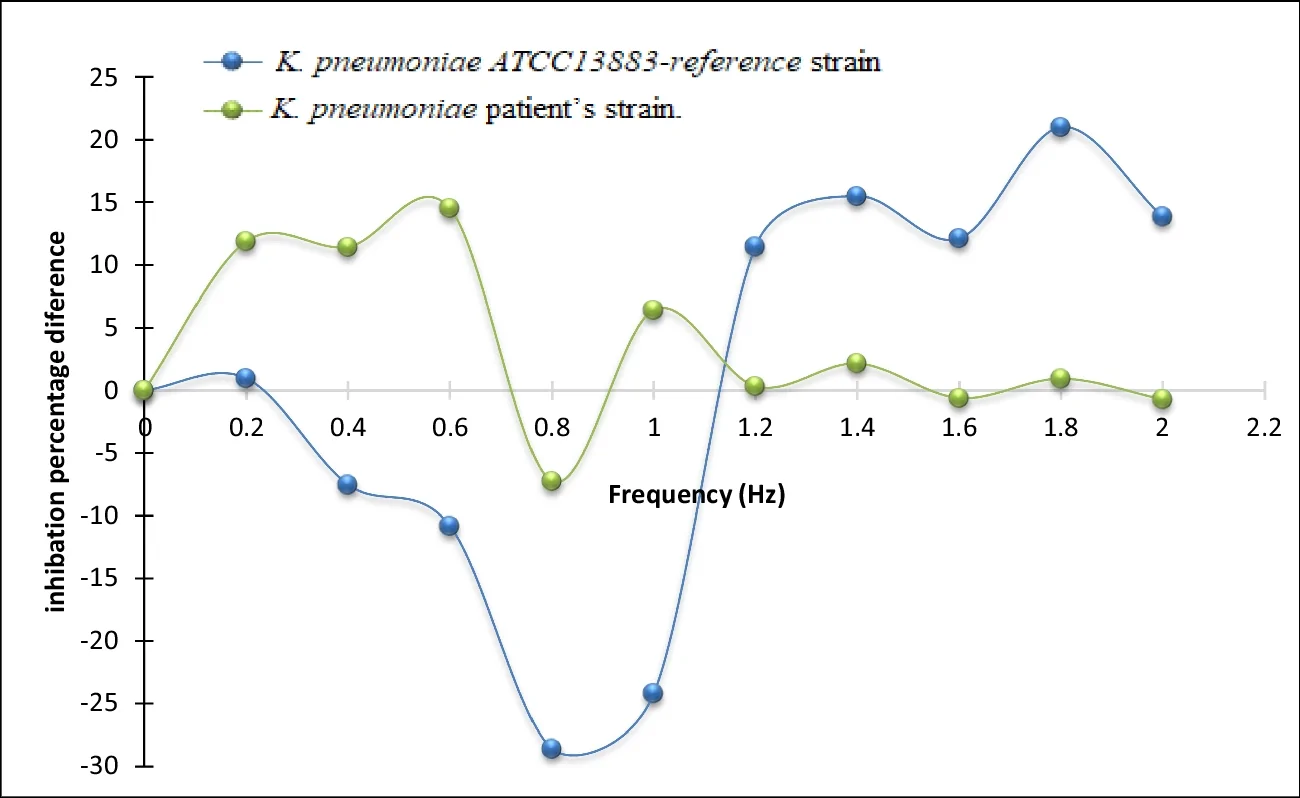
The percentage difference in inhibition between the exposed samples and the control group at different frequencies of PEF exposure for a fixed time of 30 min (after the 24th hour of incubation)

The growth curves of several samples exposed to PEF for 30 min at various frequencies were plotted at 600 nm to be compared with the control sample’s growth curve. As shown in (Fig. 1) the absorbance of the samples exposed to PEF frequencies ranging from 0.2 to 2 Hz for 30 min varied in inhibition compared to the control sample. To further investigate the inhibition resonance peak for cellular growth, the change in absorbance was measured at the 24-hour mark after incubation at different frequencies between 0.2 and 2 Hz, as shown in (Fig. 2).

Table 1 displays, following a 24-hour incubation period, the CFU/ml values for the exposed and control samples at the resonance frequencies. The results presented in Table 1 demonstrate that the growth characteristics of both strains were significantly decreased after exposure to PEF. Specifically, at a frequency of 0.8 Hz, the growth of strain A was inhibited by 96.63%, while the growth of strain B was inhibited by 96.45% after exposure for 60 min, respectively.

**Table 1:** **Table (1)** The CFU/ml value of the cells in the control and sample groups after 60 minutes (as maximum inhibition time) of exposure to 0.8 Hz PEF

Exposure Frequency/time	0.8 Hz / 60 min/(CFU/ml)		
strain	Control	Exposed	Inhibition percentage difference (D%)
K. pneumoniae ATCC13883 reference strain	959.6 ± 0.6	32.3 ± 1.9	(96.63%)
K. pneumoniae patient’s strain	929.6 ± 0.41	33 ± 2.13	(96.45%)

Significant (P < 0.001)

## Exposure time optimization at the resonance frequency

The growth curves of both the control sample and the samples subjected to a consistent frequency (0.8 Hz) of Pulsed Electric Field (PEF) for varying durations were visually represented at 600 nm for comparative analysis.

**Fig. 3:**
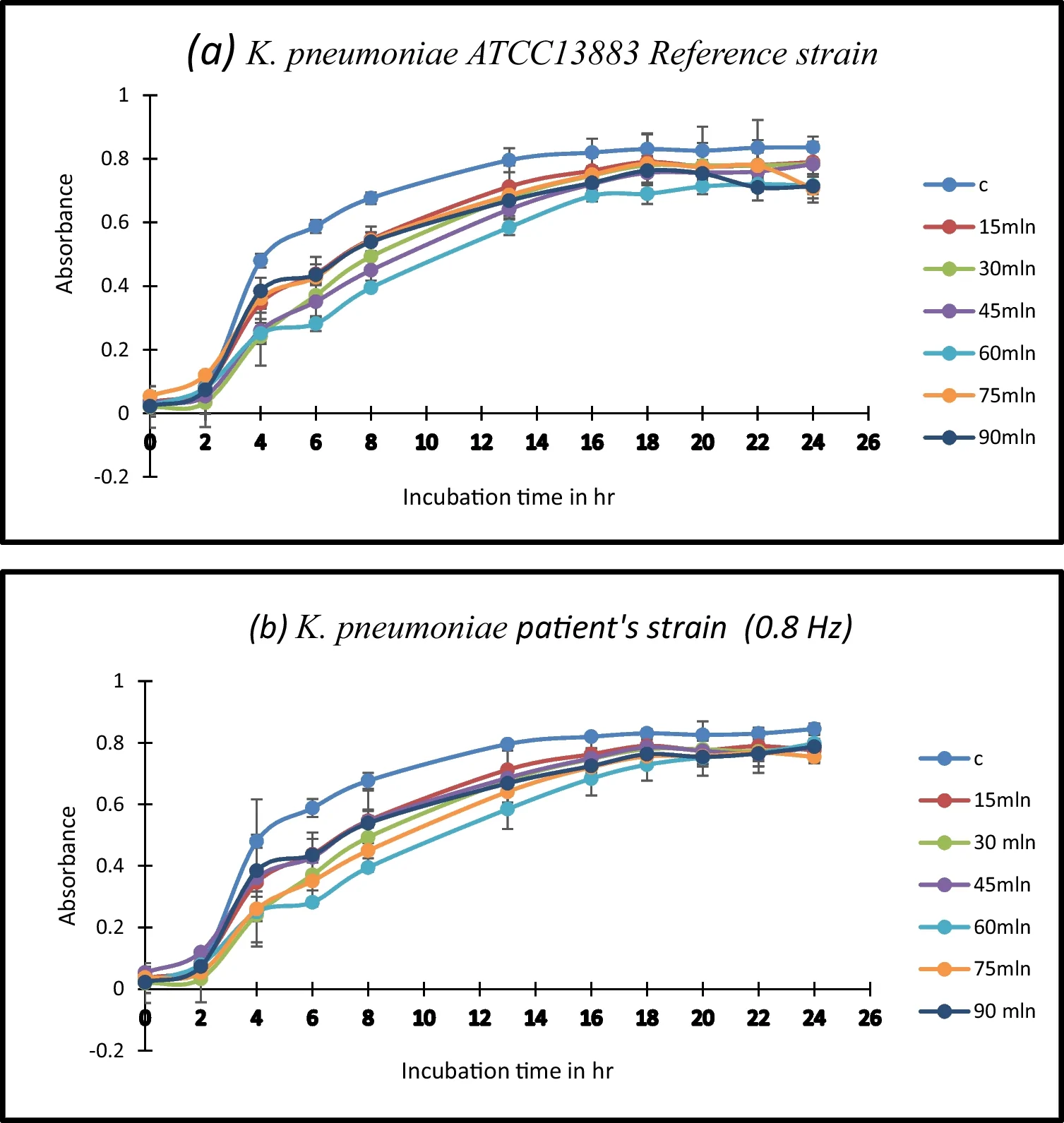
*K. Pneumoniae*’s growth curve exposed to PEF frequency 0.8 Hz at various time intervals (15–90 min) (**a**) *K. pneumoniae* ATCC13883-reference strain (**b**) *K. pneumoniae-* patient’s strain

As depicted in (Fig. 3), the absorbance values of the samples exposed to PEF across different time intervals were graphed to delve deeper into the inhibition percentage pertaining to cellular growth. This graphical representation allows for a clear visualization of how the absorbance values vary with exposure time.

**Fig. 4:**
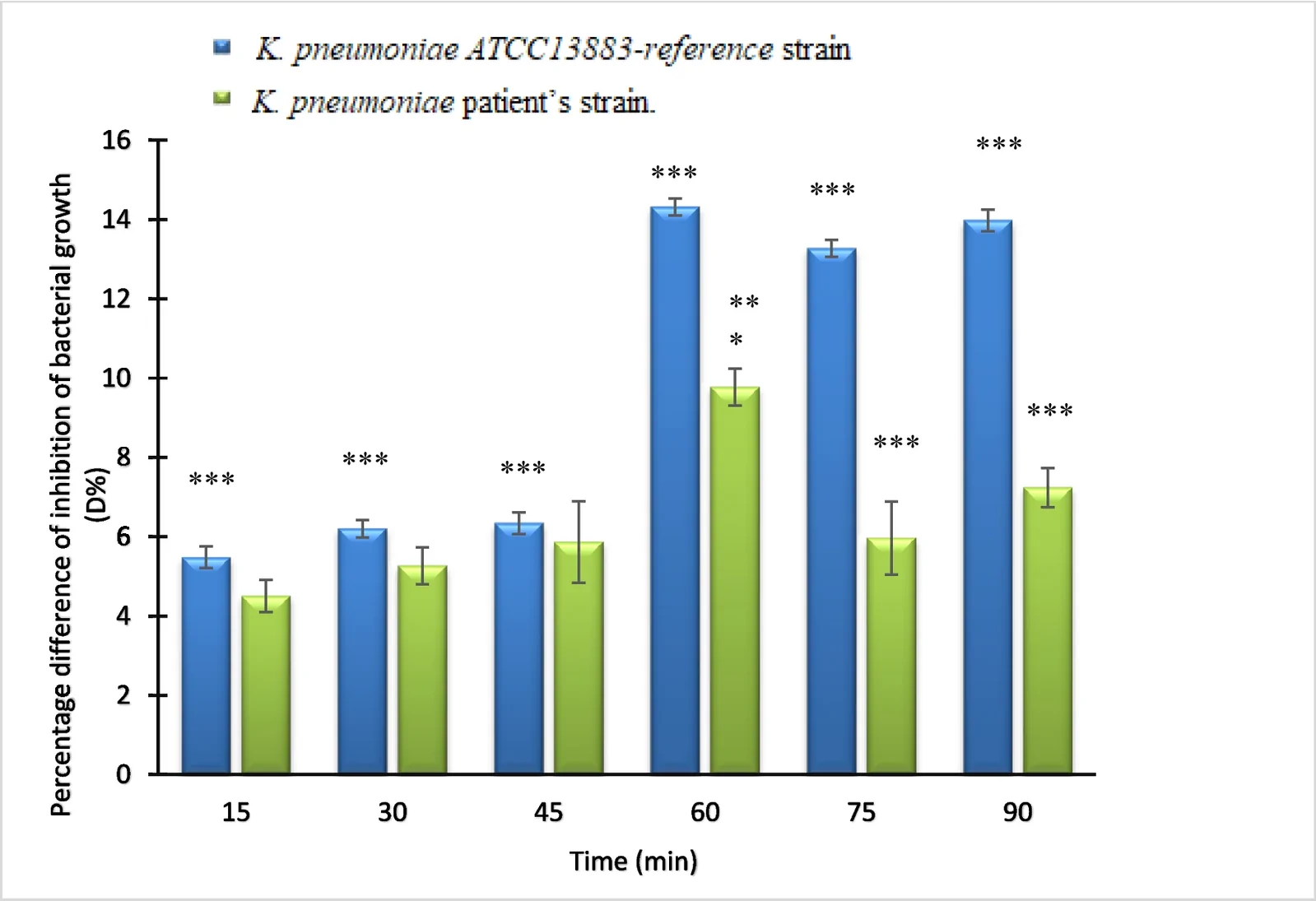
The percentage difference in inhibition between the exposed samples and the control at a PEF fixed frequency (0.8 Hz) at different time intervals. (At the 24th hour of incubation) ** significant ( P  < 0.01) and *** Significant ( P  < 0.001)

Furthermore, the alteration in absorbance was specifically assessed at the 24-hour mark, as illustrated in (Fig. 4), providing a focused view on the impact of PEF exposure duration on cellular growth inhibition. This analysis aids in understanding the effectiveness of PEF treatment in hindering cellular proliferation over a specified time period.

### The test for antibiotic susceptibility

**Fig. 5:**
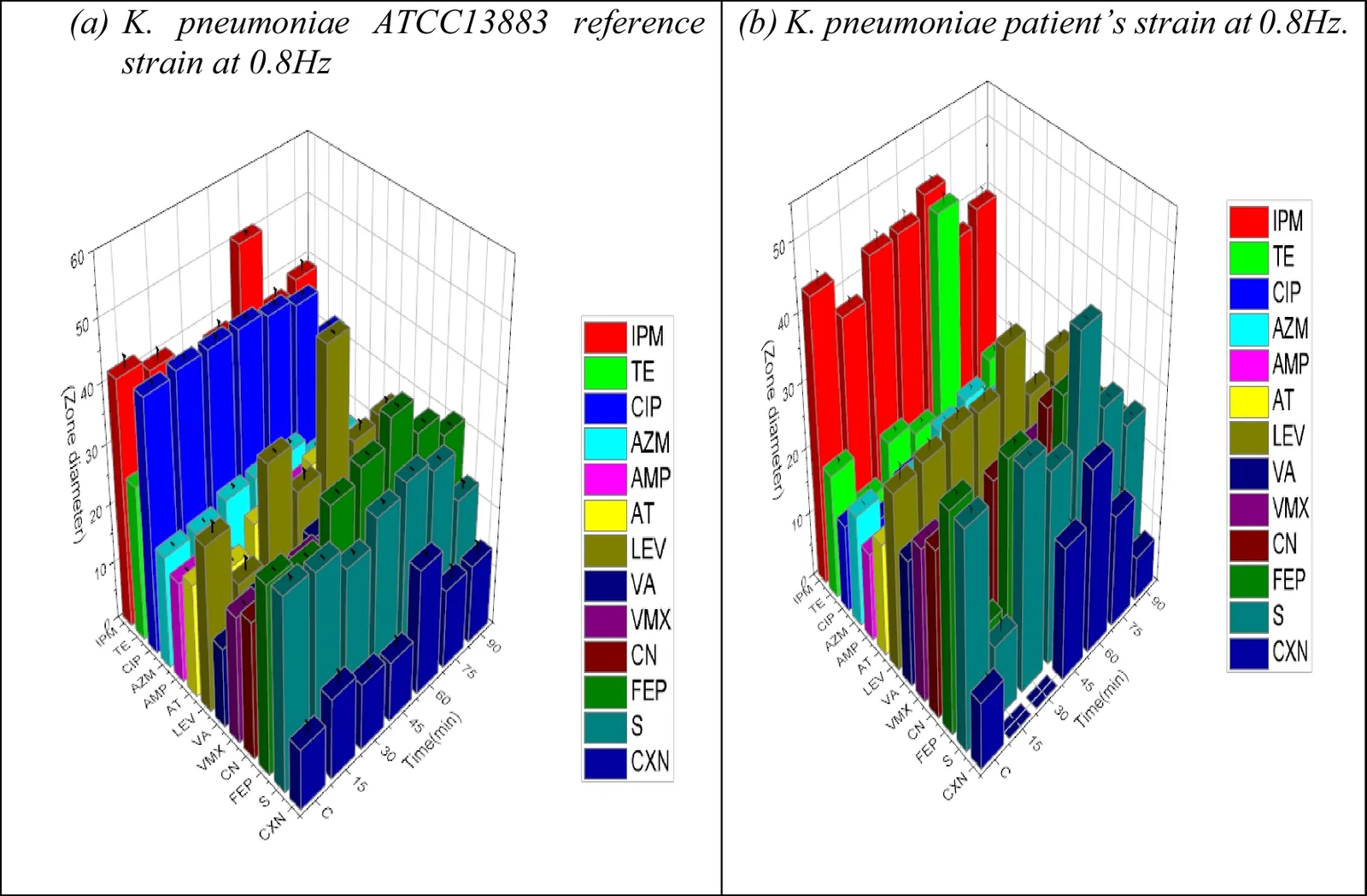
The effect of the zone diameter on the antibiotic susceptibility test at 0.8 Hz for (**a**) *K. pneumoniae* ATCC13883 reference strain and (**b**) *K. pneumoniae patient’s *strain at various exposure times. PEF (after the 24^th^ hour of incubation) uses antibiotic groups (PROTEIN INHIBITORS: Aminoglycosides S (10), Macrolides, lincosamides, and streptogramins AZM (15), Sulfonamides and synergistic agents TE (30)) ; (DNA INHIBITORS: Quinolones and fluoroquinolones CIP (5), Fluoroquinolones LEV (5), Aminoglycosides CN (10)); (β-LACTAMS AND CELL WALL INHIBITORS: Carbapenems IPM (10), Glycopeptides and lipoglycopeptides VA (30), Cephalosporins CXN (30) and FEP (30), Monobactam AT (30), β-lactams Amp(10) and AMX (10))

**Fig. 6:**
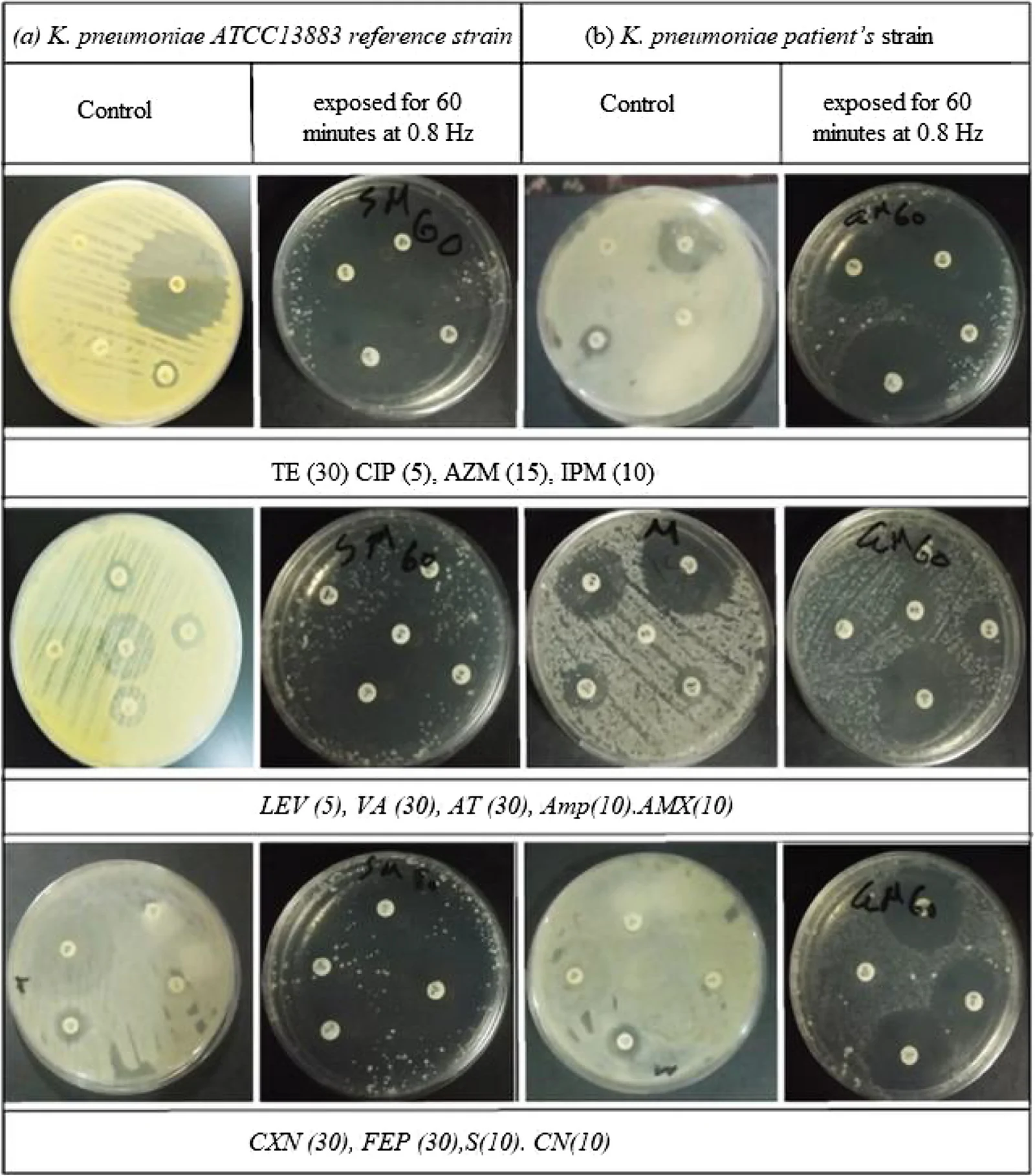
The effect of the zone diameter on the antibiotic susceptibility test at 0.8 Hz for (**a**) *K. pneumoniae* ATCC13883 reference strain and (**b**) *K. pneumoniae patient’s* strain at various exposure times. PEF (after the 24th hour of incubation) uses antibiotic groups (PROTEIN INHIBITORS: Aminoglycosides S (10), Macrolides, lincosamides, and streptogramins AZM (15), Sulfonamides and synergistic agents TE (30)) ; (DNA INHIBITORS: Quinolones and fluoroquinolones CIP (5), Fluoroquinolones LEV (5), Aminoglycosides CN (10)); (β-LACTAMS AND CELL WALL INHIBITORS: Carbapenems IPM (10), Glycopeptides and lipoglycopeptides VA (30), Cephalosporins CXN (30) and FEP (30), Monobactam AT (30), β-lactams Amp(10) and AMX (10))

The control and PEF-exposed samples differed significantly in terms of their antimicrobial susceptibility, according to the disk diffusion test results, Specifically; exposure at 0.8 Hz resulted in a substantial rise in the susceptibility to phospholipids, protein inhibitors, β-lactamase enzyme, and inhibitors of cell-wall production, as well as nucleic acids of DNA and RNA. (Figures 5 and 6) illustrate the results of the antibiotic susceptibility test, which was performed for the control and exposed samples at 0.8 Hz at various times. Different antibiotics having different biological modes of action on the microorganism were used. Results of the *K. pneumoniae* antibiotic sensitivity test are provided for control samples and samples treated to 0.8 Hz PEF, which resulted in a considerable increase in antibiotic sensitivity during 60 min.

### Biofilm formation in 96-well microtiter plates

**Fig. 7:**
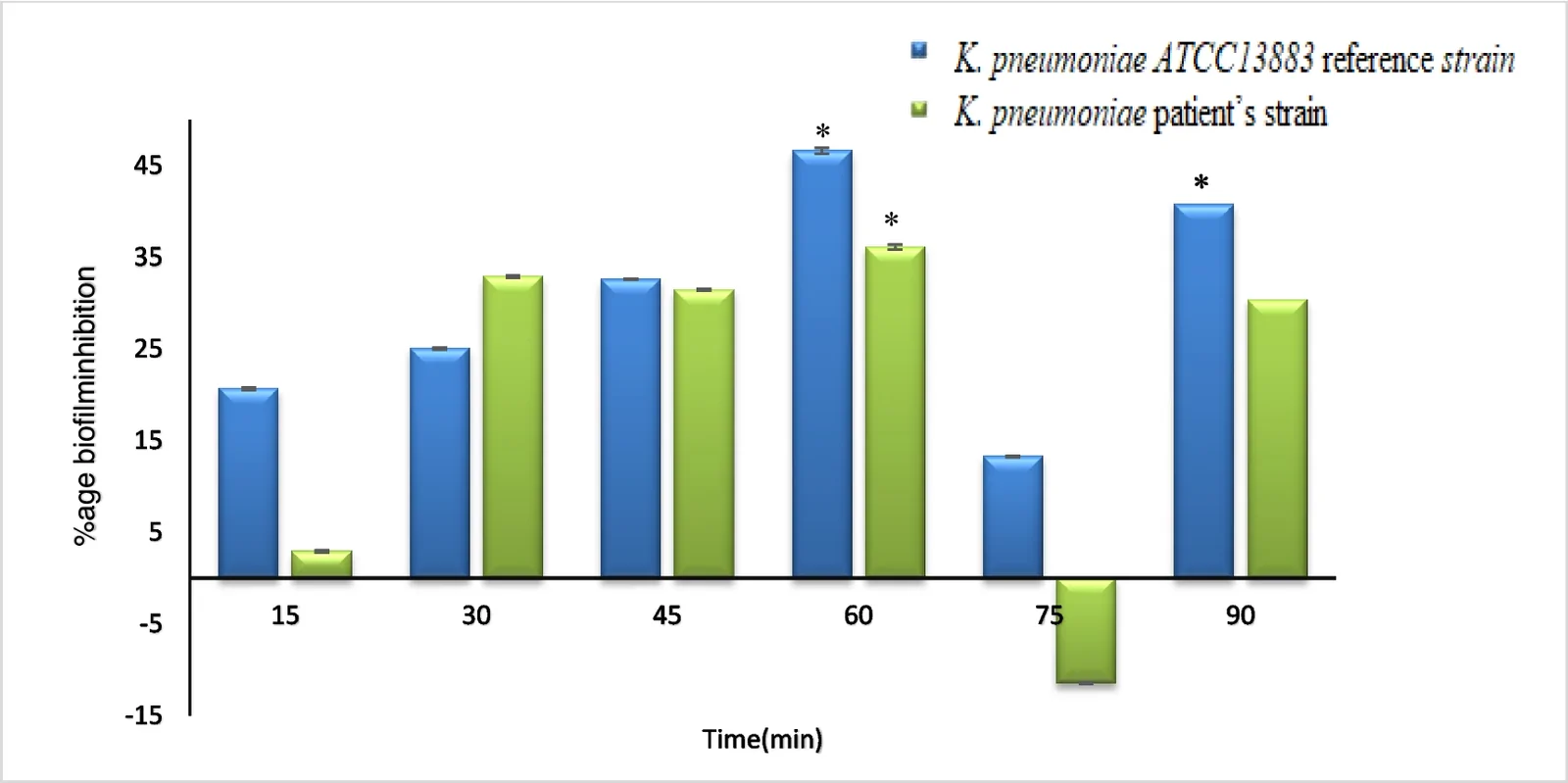
Percent inhibition of biofilm formation for the control and exposed *K. pneumoniae* samples *K. pneumoniae ATCC13883* reference strain *K. pneumoniae patient’s* strain at different times and fixed frequency PEF (0.8 Hz) ^*^Significant (*P* < 0.05)

**Fig. 8:**
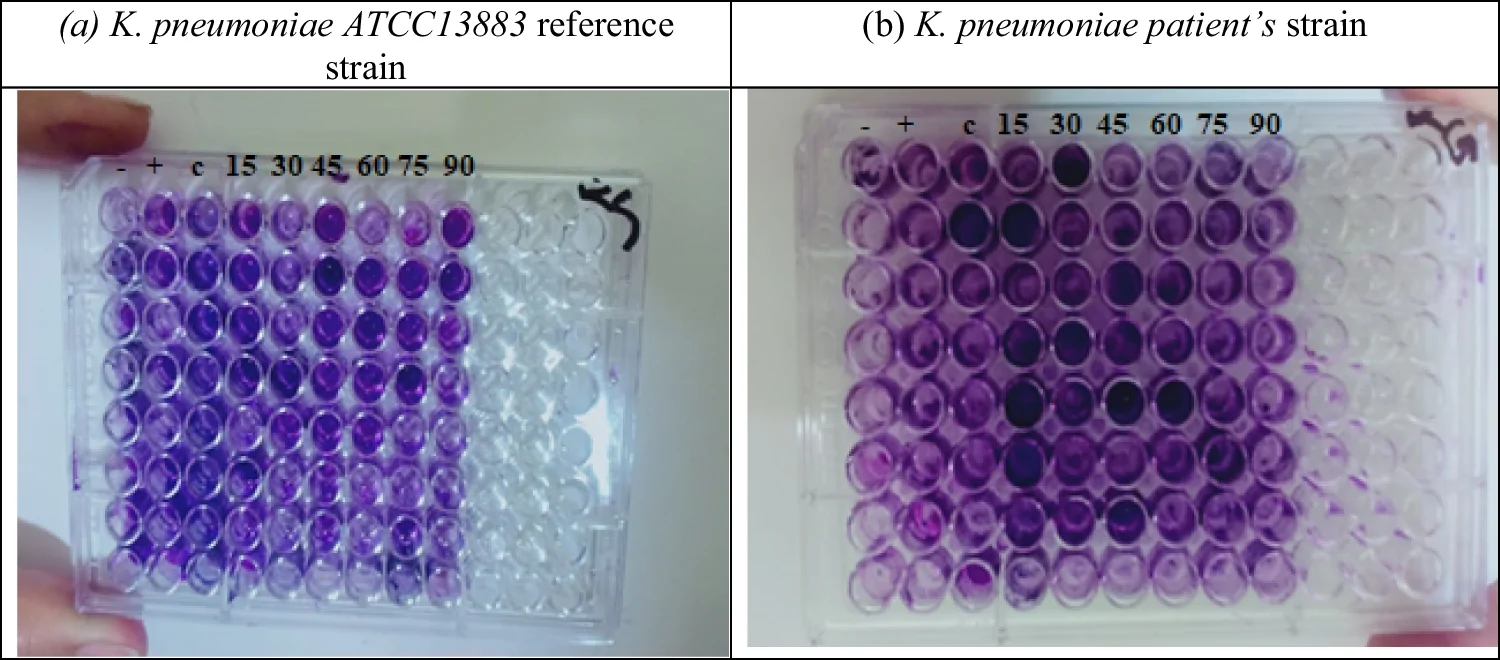
Biofilm formation by using the crystal violet microtiter plate method. (**a**) K. pneumoniae ATCC13883 reference strain (**b**) K. pneumoniae patient’s strain at different times (15–90 min) and fixed frequencies PEF (0.8 Hz) (after the 48th hour of incubation). Absorbance was measured at 630 nm

(Fig. 7) shows the absorbance of *K. pneumoniae* biofilm formation, as measured by the crystal violet assay at 630 nm. For *K. pneumoniae ATCC 13,883*, exposure to 0.8 Hz PEF for 60 min resulted in a 46.6347% increase in inhibition compared to the control sample. For the *K. pneumoniae* patient’s strain, the inhibition was 36.1133% after 60 min of exposure to 0.8 Hz PEF.

(Fig. 8) shows the 96-microtiter plate of *K. pneumoniae* biofilm formation, after staining with a crystal violet assay prepared for reading at 630 nm. Many wells contained dark violet-tinted around the perimeter of the bottom of each well even after the final wash step this indicator is for biofilm formation from the figure sample before the exposure has a maximum number of wells formed biofilm which reduced with exposure.

## Discussion

In this study, we aimed to investigate the impact of pulsed electric field (PEF) exposure at different frequencies and durations on the growth characteristics of two strains of *K. pneumoniae*. Our specific goal was to identify the resonance frequency that leads to the most significant inhibition of this pathogen’s growth. To achieve this, we analyzed various aspects of the microorganism, including its growth traits, susceptibility to antibiotics, and biofilm formation.

Previous investigation has demonstrated a strong association between the production of biofilms and the multidrug-resistant (MDR) phenotype of *K. pneumoniae* strains (Ostria-Hernandez et al. 2018). Moreover, studies have explored the relationship between the development of biofilms, antibiotic resistance, and *K. pneumoniae* strains at high concentrations, specifically sub-inhibitory levels (Maharjan et al. 2018). Furthermore, It has been determined that an outer membrane protein is essential for complement cascade inhibition and conjugation (Kuş et al. 2017). Based on previous studies (Fadel et al. 2018; Mostafa et al. 2021), we hypothesized that the outer membrane protein of *K. pneumoniae* strains could be influenced by PEF.

Earlier research (Wouters et al. 1999; Ali et al. 2013, 2017; Fadel et al. 2017) has demonstrated that cells and organs generate bioelectric signals within the extremely low-frequency range during their metabolic processes. Building upon these findings, According to our research, the applied PEF wave should have a similar frequency to interfere with these signals. We evaluated the growth curves of a variety of samples that were exposed to PEF for 30 min at different frequencies to test this theory and identify the resonance frequency. The reference and patient strains of *K. pneumoniae* growth curves were analyzed, and the results showed that the highest inhibition was obtained at a frequency of 0.8 Hz PEF. Furthermore, According to our data, samples subjected to PEF at the resonant frequency for 60 min had a significant (*P* < 0.001) decrease in population intensity, which resulted in maximal growth inhibition of 96.63% and 96.45%, respectively, for both strains.

It is well known that ionic mobility, which produces ionic currents and potentials, is a component of the metabolic processes of live biological systems. These bioelectric potentials’ waveform and frequency serve as a distinctive system fingerprint, representing the physiological processes at work. These bioelectric currents produce bio-magnetic fields that have the potential to interfere with adjacent cells’ ability to metabolize (Ali et al. 2013, 2016). Our findings are consistent with other research (Chen et al. 2019; Mostafa et al. 2021, 2023) that documented the inhibitory effects of electric fields on *K. pneumoniae* growth and viability as well as biofilm formation. The alterations in the packing characteristics of phospholipid macromolecules, which constitute the cellular membrane and influence membrane permeability and ionic pumping mechanisms, could be connected to the variations in electrostatic surface charges of exposed cells. Antibiotic sensitivity test findings, which employ a variety of medicines with distinct modes of interaction with the microorganism, clearly demonstrate this occurrence. Changes in the bacterial uptake process of aminoglycosides, which are cationic molecules that bind to anionic components of the bacterial cell membrane in a reversible and concentration-dependent manner may be the cause of the increased susceptibility to protein synthesis inhibitors (Taber et al. 1987).

Similarly, PEF’s impact on mucopeptide production in the bacterial cell wall may account for the microorganism’s heightened vulnerability to inhibitors of cell wall synthesis following exposure. Notably, for both patient and reference strains, the disc diffusion approach revealed the highest effect of exposure (0.8 Hz, 60 min), which was very significant (*P* < 0.001) when compared to other exposure periods. The observed distinction between the clinically separated strain and the reference strain can be ascribed to established processes of niche specialization and host adaptation.

Enterobacteriaceae pathogens and commensals are directly transferred from person to person and come into touch with more antibiotics as the population grows. Hospital infections are often linked to K. *pneumoniae*, a major cause of sepsis in humans (Podschun et al., 1998; Fajardo et al., 2019). Our results are in line with other research that has documented the inhibitory effects of extremely low-frequency electromagnetic fields (ELF-EMFs), which vary depending on the length of exposure, on the growth and viability of heterotrophic bacteria (Fadel et al. 2018; Mostafa et al. 2021).

To investigate the effect of PEF on biofilm formation, we exposed bacterial cultures in microtiter plates to 0.8 Hz PEF at different durations, comparing them to non-exposed controls. The results showed mostly inhibition of biofilm development after one hour, although there was a significant (*P* < 0.05) stimulation of biofilm development at 30 min of exposure for the reference strain. This stimulation could be due to the initial disruption caused by PEF exposure, leading to an adaptive response by the bacteria to enhance biofilm formation as a protective mechanism.

In conclusion, our study demonstrates that PEF exposure at a specific resonance frequency can significantly inhibit the growth of *K. pneumoniae* strains. The inhibitory effect is observed in terms of growth characteristics, antibiotic susceptibility, and biofilm formation. These findings suggest that PEF treatment has the potential to be used as a novel approach for controlling *K. pneumonia* infections. Further research is needed to explore the underlying mechanisms and optimize the PEF parameters for maximum efficacy and safety in clinical settings.

## Electronic supplementary material

Below is the link to the electronic supplementary material.

Supplementary Material 1

## Data Availability

The corresponding author can provide the datasets created and/or analyzed during the current work upon justifiable request.

## References

[CR1] Al-Agha AGM, Al-Khafaji NJM, Al-Azawi AKS (2017) Isolation and identification of *Klebsiella pneumoniae* using API-20E analytical system and conventional PCR assay. Int J Curr Microbiol App Sci 6(8):203–210. 10.20546/ijcmas.2016.501.028

[CR2] Ali FM, Elkhatib AM, Aboutalib WM, Abdelbacki AM, Khalil AM, Serag N (2013) Control of the activity of *Pseudomonas aeruginosa* by positive electric impulses at resonance frequency. J Am Sci 9(10):120–130. http://www.jofamericanscience.org

[CR3] Ali FM, Elgebaly RH, Elneklawi MS, Othman AS (2016) Role of duty cycle on *Pseudomonas aeruginosa* growth inhibition mechanisms by positive electric pulses. Bio-med Mater Eng 27(2–3):211–225. 10.3233/BME-161577 10.3233/BME-161577 27567776

[CR4] Ali FM, El-Gebaly RH, Hamad AM (2017) Combination of bacteriolytic therapy with magnetic field for Ehrlich tumour treatment. Gen Physiol Biophys 36(3):259–271. 10.4149/gpb_2016051 28471345 10.4149/gpb_2016051

[CR5] Askaripour K, Żak A (2024) A systematic review on cellular responses of Escherichia coli to nonthermal electromagnetic irradiation. Bioelectromag 45(1):16–29. 10.1002/bem.22484 10.1002/bem.22484 37807247

[CR6] Berrazeg M, Diene SM, Drissi M, Kempf M, Richet H, Landraud L, Rolain JM (2013) Biotyping of multidrug-resistant *Klebsiella pneumoniae* clinical isolates from France and Algeria using MALDI-TOF MS. PLoS ONE 8(4):e61428. 10.1371/journal.pone.0061428 23620754 10.1371/journal.pone.0061428 PMC3631213

[CR7] Boisvert AA, Cheng MP, Sheppard DC, Nguyen D (2016) Microbial biofilms in pulmonary and critical care diseases. Annals Am Thorac Soc 13(9):1615–1623. 10.1513/AnnalsATS.201603-194FR 10.1513/AnnalsATS.201603-194FR PMC5059503 27348071

[CR8] Bouhrour N, Nibbering PH, Bendali F (2024) Medical device-Associated Biofilm infections and Multidrug-resistant pathogens. Pathogens (Basel Switzerland) 13(5):393. 10.3390/pathogens13050393 38787246 10.3390/pathogens13050393 PMC11124157

[CR9] Chang UI, Kim HW, Wie SH (2015) Propensity-matched analysis to compare thetherapeutic efficacies of cefuroxime versus cefotaxime as initial antimicrobial therapy for community-onset complicated nonobstructive acute pyelonephritis due to Enterobacteriaceae infection in women. Antimicrob Agents Chemother 59(5):2488–2495 10.1128/AAC.04421-14 PMC4394823 25645837

[CR10] Chen Y, Cai Z, Feng Q, Gao P, Yang Y, Bai X, Tang BQ (2019) Evaluation of the extremely low-frequency electromagnetic field (ELF-EMF) on the growth of bacteria *Escherichia Coli*. Cogent Biol 5(1):1625104. 10.1080/23312025.2019.1625104

[CR11] Cubero M, Marti S, Domínguez MÁ, González-Díaz A, Berbel D, Ardanuy C (2019) Hypervirulent *Klebsiella pneumoniae* serotype K1 clinical isolates form robust biofilms at the air-liquid interface. PLoS ONE 14(9):e0222628. 10.1371/journal.pone.0222628 31532800 10.1371/journal.pone.0222628 PMC6750583

[CR12] Da Silva FA Jr, Alcaraz-Espinoza JJ, da Costa MM, de Oliveira HP (2019) Low intensity electric field inactivation of gram-positive and gram-negative bacteria via metal-free polymeric composite. Mater Sci Engineering: C 99:827–837 10.1016/j.msec.2019.02.027 30889757

[CR13] David S, Reuter S, Harris SR, Glasner C, Feltwell T, Argimon S, Abudahab K, Goater R, Giani T, Errico G, Aspbury M, Sjunnebo S, EuSCAPE Working Group, ESGEM Study Group, Feil EJ, Rossolini GM, Aanensen DM, Grundmann H (2019) Epidemic of carbapenem-resistant *Klebsiella pneumoniae* in Europe is driven by nosocomial spread. Nat Microbiol 4(11):1919–1929. 10.1038/s41564-019-0492-8 31358985 10.1038/s41564-019-0492-8 PMC7244338

[CR14] Di Domenico EG, Cavallo I, Sivori F, Marchesi F, Prignano G, Pimpinelli F, Sperduti I, Pelagalli L, Di Salvo F, Celesti I, Paluzzi S, Pronesti C, Koudriavtseva T, Ascenzioni F, Toma L, De Luca A, Mengarelli A, Ensoli F (2020) Biofilm production by carbapenem-resistant *Klebsiella pneumoniae* significantly increases the risk of death in oncological patients. Front Cell Infect Microbiol 10561741. 10.3389/fcimb.2020.561741 10.3389/fcimb.2020.561741 PMC7759150 33363047

[CR15] El-Kholy AA, Girgis SA, Shetta MA, Abdel-Hamid DH, Elmanakhly AR (2020) Molecular characterization of multidrug-resistant Gram-negative pathogens in three tertiary hospitals in Cairo, Egypt. Eur J Clin Microbiol Infect Dis 39(5):987–992. 10.1007/s10096-020-03812-z 31953591 10.1007/s10096-020-03812-z PMC7182536

[CR16] Elsaid EM, Ahmed OI, Abdo AM, Abdel Salam SA (2023) Antimicrobial and antibiofilm effect of silver nanoparticles on clinical isolates of multidrug resistant *Klebsiella Pneumoniae*. Microb Infect Dis 4(2):542–554. 10.21608/MID.2023.200483.1487

[CR17] Fadel MA, Mohamed SA, Abdelbacki AM, El-Sharkawy AH (2014) Inhibition of *Salmonella typhi* growth using extremely low frequency electromagnetic (ELF‐EM) waves at resonance frequency. J Appl Microbiol 117(2):358–365. 10.1111/jam.12527 24766529 10.1111/jam.12527

[CR18] Fadel MA, El-Gebaly RH, Mohamed SA, Abdelbacki AM (2017) Biophysical control of the growth of *Agrobacterium tumefaciens* using extremely low-frequency electromagnetic waves at resonance frequency. Biochem Biophys Res Commun 494(1–2):365–371. 10.1016/j.bbrc.2017.10.008 28988110 10.1016/j.bbrc.2017.10.008

[CR19] Fadel MA, Mohamed ZA, Abdellateef MA, Hosny AA (2018) Effect of extremely low frequency of electromagnetic fields on some toxic species of cyan bacteria. Inter J New Horizon Phys 5(1):5–10. 10.18576/ijnhp/050102

[CR20] Fajardo-Lubián A, Ben Zakour NL, Agyekum A, Qi Q, Iredell JR (2019) Host adaptation and convergent evolution increases antibiotic resistance without loss of virulence in a major human pathogen. PLoS Pathog 15(3):e1007218. 10.1371/journal.ppat.1007218 30875398 10.1371/journal.ppat.1007218 PMC6436753

[CR21] Guzmán-Armenteros TM, Villacís-Chiriboga J, Guerra LS, Ruales J (2024) Electromagnetic fields effects on microbial growth in cocoa fermentation: a controlled experimental approach using established growth models. Heliyon 10(3):e24927. 10.1016/j.heliyon.2024.e24927 38317962 10.1016/j.heliyon.2024.e24927 PMC10839996

[CR22] Humphries R, Bobenchik AM, Hindler JA, Schuetz AN (2021) Overview of changes to the clinical and laboratory standards institute performance standards for antimicrobial susceptibility testing, M100. J Clin Microbiol 59(12):e0021321. 10.1128/JCM.00213-21 34550809 10.1128/JCM.00213-21 PMC8601225

[CR23] Inhan-Garip A, Aksu B, Akan Z, Akakin D, Ozaydin AN, San T (2011) Effect of extremely low frequency electromagnetic fields on growth rate and morphology of bacteria. Int J Radiat Biol 87(12):1155–1161. 10.3109/09553002.2011.560992 21401315 10.3109/09553002.2011.560992

[CR24] Joseph BJ, Mathew M, Rachel R, Mathew J, Radhakrishnan EK (2024). *Klebsiella pneumoniae* Virulence Factors and Biofilm Components: Synthesis, Structure, Function, and Inhibitors. In: Busi, S., Prasad, R. (eds) ESKAPE Pathogens. Springer, Singapore. 10.1007/978-981-99-8799-3_9

[CR25] Karimi K, Zarei O, Sedighi P, Taheri M, Doosti-Irani A, Shokoohizadeh L (2021) Investigation of antibiotic resistance and biofilm formation in clinical isolates of *Klebsiella pneumoniae*. Int J Microbiol 5573388. 10.1155/2021/5573388 10.1155/2021/5573388 PMC8219462 34221021

[CR26] Khan E, Ejaz M, Zafar A, Jabeen K, Shakoor S, Inayat R, Hasan R (2010) Increased isolation of ESBL producing *Klebsiella pneumoniae* with emergence of carbapenem resistant isolates in Pakistan: report from a tertiary care hospital. J Pak Med Assoc 60(3):186–190 PMID: 20225774 20225774

[CR27] Kurokawa I, Kanayama S, Yamasaki O (2022) Antimicrobial activity of ozenoxacin and other antimicrobials against *Staphylococcus aureus* strains isolated from clinical skin specimens in Japan in 2019 and 2020. J Infect Chemother 28(12):1693–1696. 10.1016/j.jiac.2022.08.014 35988886 10.1016/j.jiac.2022.08.014

[CR28] Kuş H, Arslan U, Türk Dağı H, Fındık D (2017) Investigation of various virulence factors of *Klebsiella pneumoniae* strains isolated from nosocomial infections. Mikrobiyol Bul 51(4):329–339. 10.5578/mb.59716 29153063 10.5578/mb.59716

[CR29] Kwiatkowski P, Sienkiewicz M, Pruss A, Łopusiewicz Ł, Arszyńska N, Wojciechowska-Koszko I, Kilanowicz A, Kot B, Dołęgowska B (2022) Antibacterial and anti-biofilm activities of essential oil compounds against New Delhi Metallo-β-lactamase-1-producing uropathogenic *Klebsiella pneumoniae* strains. Antibiotics 11(2):147. 10.3390/antibiotics11020147 35203751 10.3390/antibiotics11020147 PMC8868355

[CR30] Maharjan G, Khadka P, Siddhi Shilpakar G, Chapagain G, Dhungana GR (2018) Catheter-associated urinary tract infection and obstinate biofilm producers. Can J Infect Dis Med Microbiol 2018: 7624857. 10.1155/2018/7624857 10.1155/2018/7624857 PMC6129315 30224941

[CR31] Mirghani R, Saba T, Khaliq H, Mitchell J, Do L, Chambi L, Diaz K, Kennedy T, Alkassab K, Huynh T, Elmi M, Martinez J, Sawan S, Rijal G (2022) Biofilms: formation, drug resistance and alternatives to conventional approaches. AIMS Microbiol Jul 4(3):239–277. 10.3934/microbiol.2022019PMID: 36317001; PMCID: PMC9576500 10.3934/microbiol.2022019 PMC9576500 36317001

[CR32] Monod J (2012) The growth of bacterial cultures. Sel Papers Mol Biology Jacques Monod 139:606

[CR33] Mostafa MR, Ali F, Balabel NM (2021) Electric pulses decrease the growth activity of *Erwinia amylovora bacterium*. Afr Biol Sci 17(1):261–270. 10.21608/ajbs.2021.201678

[CR34] Mostafa MM, Mohamad EA, Ramadan MA, Elneklawi MS (2023) Reduced Graphene Oxide@ Magnetite Nanocomposite and ELFEF effect on *Staphylococcus aureus* growth inhibition. Egyp J Chem 66(6):267–278. 10.21608/ejchem.2022.157530.6825

[CR35] Nirwati H, Sinanjung K, Fahrunissa F, Wijaya F, Napitupulu S, Hati VP, Hakim MS, Meliala A, Aman AT, Nuryastuti T (2019) Biofilm formation and antibiotic resistance of *Klebsiella pneumoniae* isolated from clinical samples in a tertiary care hospital, Klaten. Indonesia. BMC Proc. 13(11):20. 10.1186/s12919-019-0176-7 31890013 10.1186/s12919-019-0176-7 PMC6913045

[CR36] Oncul S, Cuce EM, Aksu B, Inhan Garip A (2016) Effect of extremely low frequency electromagnetic fields on bacterial membrane. Int J Radiat Biol 92(1):42–49. 10.3109/09553002.2015.1101500 26514970 10.3109/09553002.2015.1101500

[CR37] Ostria-Hernandez ML, Juárez-de la Rosa KC, Arzate-Barbosa P, Lara-Hernández A, Sakai F, Ibarra JA, Castro-Escarpulli G, Vidal JE (2018) Nosocomial, multidrug-resistant *Klebsiella pneumoniae* strains isolated from Mexico city produce robust biofilms on abiotic surfaces but not on human lung cells. Microb Drug Resist 24(4):422–433. 10.1089/mdr.2017.0073 28915364 10.1089/mdr.2017.0073 PMC5946738

[CR38] Panickar A, Manoharan A, Anbarasu A, Ramaiah S (2024) Respiratory tract infections: an update on the complexity of bacterial diversity, therapeutic interventions and breakthroughs. Arch Microbiol 206:382. 10.1007/s00203-024-04107-z 39153075 10.1007/s00203-024-04107-z

[CR39] Podschun R, Ullmann U (1998) *Klebsiella spp.* as nosocomial pathogens: epidemiology, taxonomy, typing methods, and pathogenicity factors. Clin Microbio Rev 11(4):589–603. 10.1128/CMR.11.4.589 10.1128/cmr.11.4.589 PMC88898 9767057

[CR40] Segatore B, Setacci D, Bennato F, Cardigno R, Amicosante G, Iorio R (2012) Evaluations of the effects of extremely low-frequency electromagnetic fields on growth and antibiotic susceptibility of *Escherichia coli* and *Pseudomonas aeruginosa*. Int J Microbiol 2012: 587293. 10.1155/2012/587293 10.1155/2012/587293 PMC3335185 22577384

[CR41] Serag N (2013) : Genetic changes associating exposure of microorganisms to both enhancing or inhibiting electromagnetic waves and its application (Unpublished doctoral dissertation). Cairo University, Egypt

[CR42] Serag N, Fadel MA, Osoris WG (2014) : Healing of injuries of guinea pig contaminated with Pseudomonas aeruginosa by 0.7 Hz square magnetic impulses (new method). Conference: Proceedings of the German Biophysical Society. (Germany) Sep. 14 – Sep. 17, 2014

[CR43] Singh S, Singh SK, Chowdhury I, Singh R (2017) Understanding the mechanism of bacterial biofilms resistance to antimicrobial agents. open Microbiol J 11:53 28553416 10.2174/1874285801711010053 PMC5427689

[CR44] Skinner FA, Jones PC, Mollison JE (1952) A comparison of a direct- and a plate counting technique for the quantitative estimation of soil micro-organisms. J Gen Microbiol 6(3–4):261–271. 10.1099/00221287-6-3-4-261 14927873 10.1099/00221287-6-3-4-261

[CR45] Stepanovic S, Vukovic D, Dakic I, Savic B, Svabic-Vlahovic M (2000) A modifiedmicrotiter-plate test for quantification of staphylococcal biofilm formation. J Microbiol Methods 40(2):175–179. 10.1016/s0167-7012(00)00122-6 10.1016/s0167-7012(00)00122-6 10699673

[CR46] Taber HW, Mueller JP, Miller PF, Arrow AS (1987) Bacterial uptake of aminoglycoside antibiotics. Microbiol Rev 51(4):439–457. 10.1128/mr.51.4.439-457.1987 3325794 10.1128/mr.51.4.439-457.1987 PMC373126

[CR47] Versalovic J (ed) (2011) Manual of clinical microbiology, vol 1. American Society for Microbiology

[CR48] Wang B, Wei PW, Wan S, Yao Y, Song CR, Song PP, Xu GB, Hu ZQ, Zeng Z, Wang C, Liu HM (2021) Ginkgo biloba exocarp extracts inhibit *S. Aureus* and MRSA by disrupting biofilms and affecting gene expression. J Ethnopharmacol 271113895. 10.1016/j.jep.2021.113895 10.1016/j.jep.2021.113895 33524512

[CR49] Woroszyło M, Ciecholewska-Juśko D, Junka A, Drozd R, Wardach M, Migdał P, Szymczyk-Ziółkowska P, Styburski D, Fijałkowski K (2021) Rotating magnetic field increases β-Lactam Antibiotic susceptibility of Methicillin-resistant *Staphylococcus aureus* strains. Int J Mol Sci 22(22):12397. 10.3390/ijms222212397 34830278 10.3390/ijms222212397 PMC8618647

[CR50] Wouters PC, Dutreux N, Smelt JP, Lelieveld HL (1999) Effects of pulsed electric fields on inactivation kinetics of *Listeria innocua*. Appl Environ Microbiol 65(12):5364–5371. 10.1128/AEM.65.12.5364-5371.1999 10583990 10.1128/aem.65.12.5364-5371.1999 PMC91730

