# Supplementary material for: Pulsed electric field at resonance frequency combat Klebsiella pneumonia biofilms
Source: Appl Microbiol Biotechnol. 2024 Nov 5;108:505. doi: 10.1007/s00253-024-13330-z (PMC11538251; doi:10.1007/s00253-024-13330-z)
Supplement: Supplementary file 1 [file 253_2024_13330_MOESM1_ESM.pdf]

Journal Name: Applied Microbiology and Biotechnology

Article title: Pulsed Electric Field at Resonance Frequency Combat Klebsiella pneumonia Biofilms

Authors Names: Dorria H. Mohamed[1], Haitham S. Mohammed[1*], Reem H. El-Gebaly[1], Mohamed Adam[2], Fadel M. Ali[1]

Affiliations: [1]Biophysics Department, Faculty of Science, Cairo University, Giza, Egypt. [2]Agricultural Zoology and Nematology Department, Faculty of Agriculture, Cairo University, Egypt.

***Corresponding author:**
Haitham S. Mohammed, PhD
Biophysics Department
Faculty of Science
Cairo University
Giza, Egypt
E-mail: Haitham@sci.cu.edu.eg
Tel: +201228431709
Google Scholar: https://scholar.google.com/citations?user=wCJhVWoAAAAJ&hl=en
Scopus ID: 46061314100
ORCID: https://orcid.org/0000-0002-6055-9514

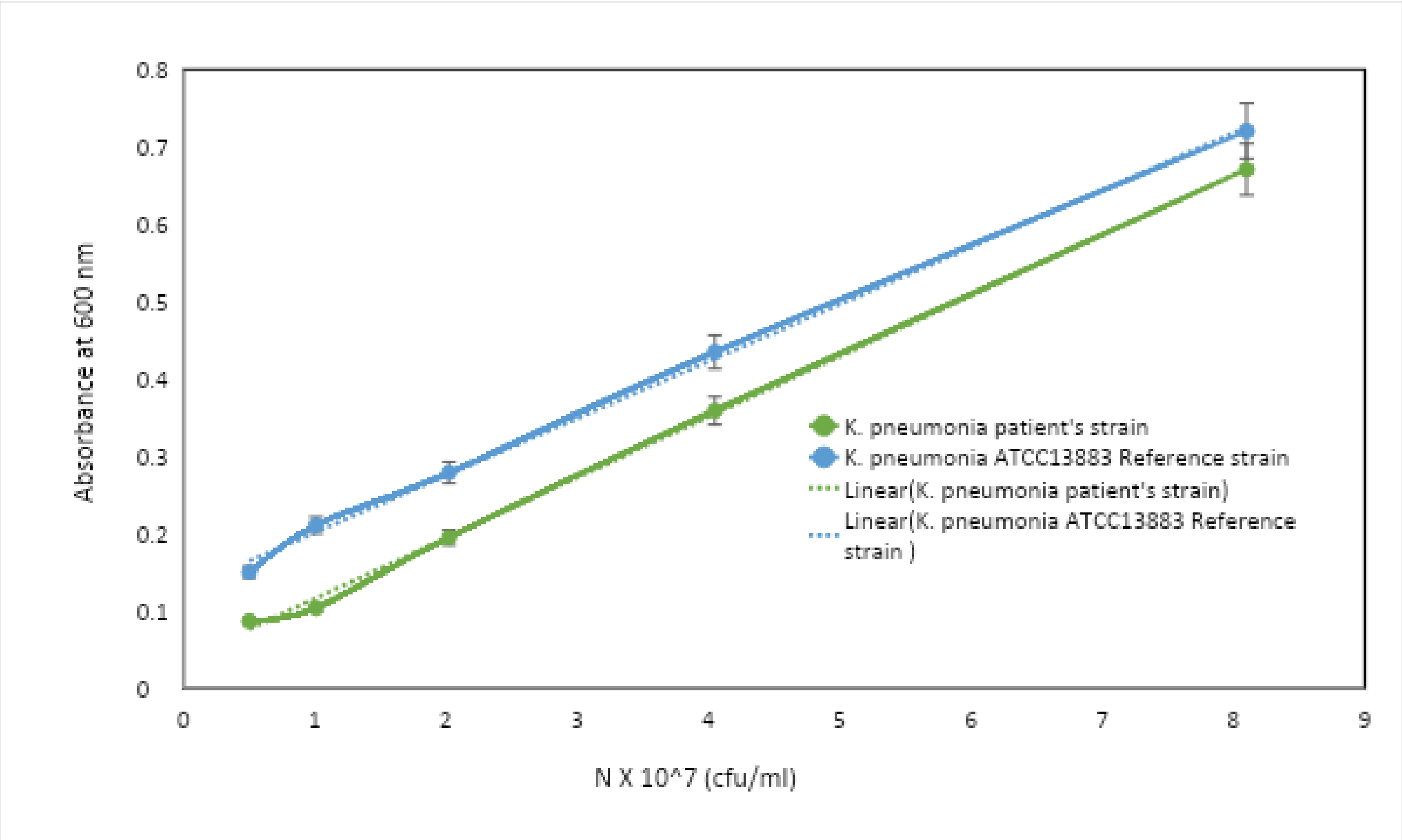

Fig.S1 Count-absorbance calibration curve for *K. pneumoniae* bacteria.

PEF exposure system and experimental design

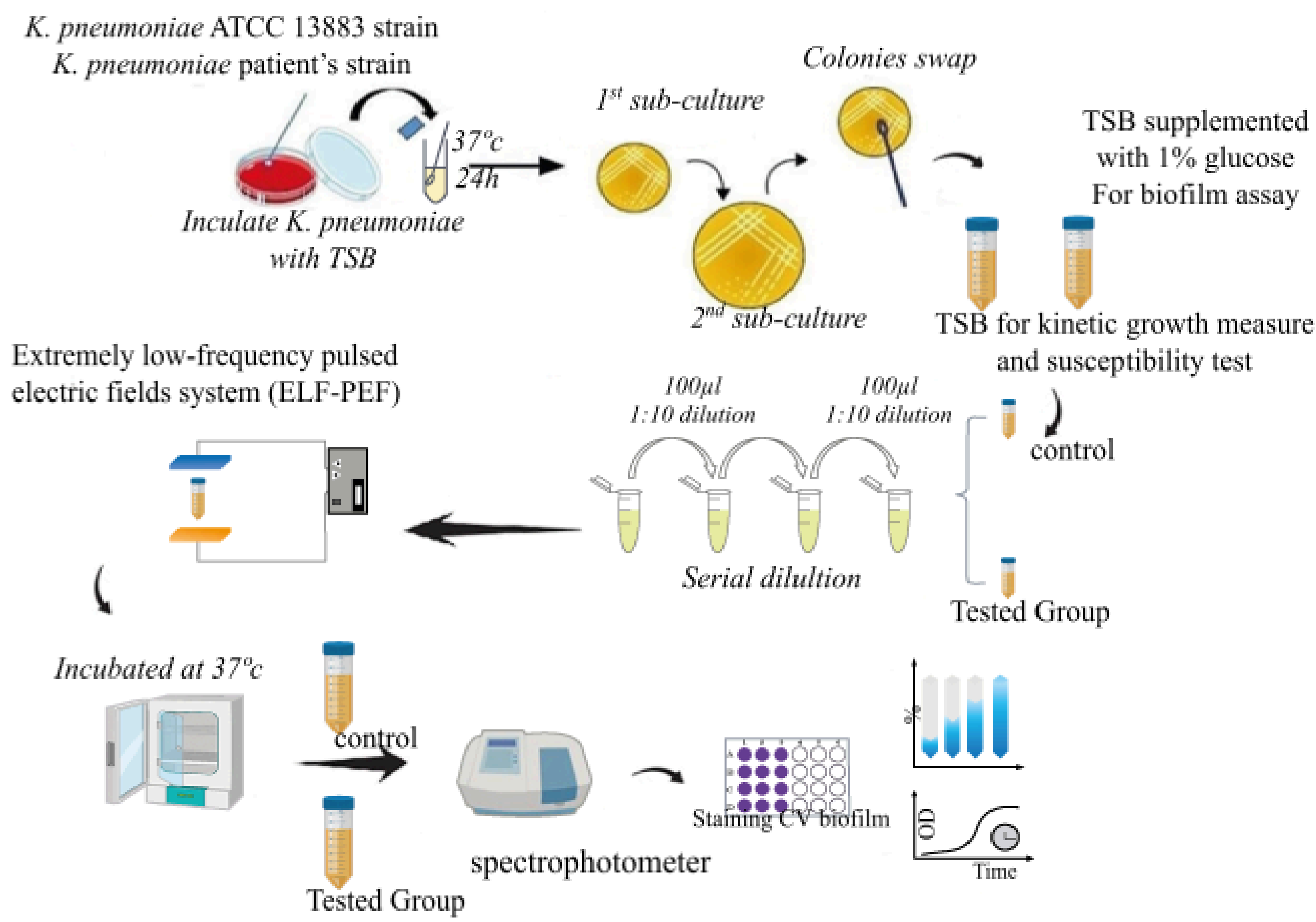

Fig.S2 Diagrammatic representation of the experimental design and exposure configuration.

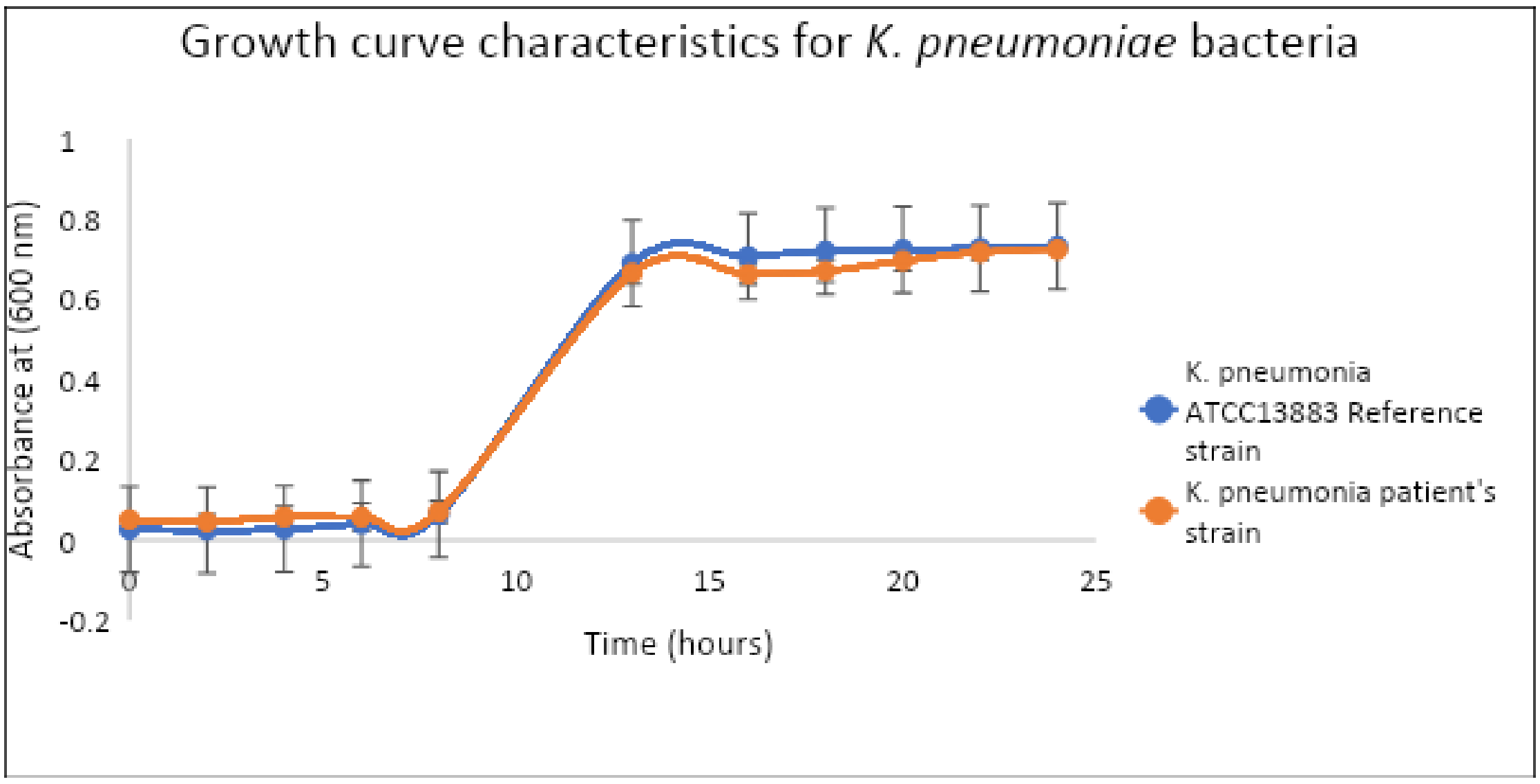

Fig.S3 Growth curve characteristics for *K. pneumonia* bacteria. optical density determinations at 600 nm, versus time of incubation (hours). Data are the mean of the optical density of each tube (n = 4) ± SD
